# The application of exosomes in the treatment of triple-negative breast cancer

**DOI:** 10.3389/fmolb.2022.1022725

**Published:** 2022-11-10

**Authors:** John W. Weaver, Jinyu Zhang, Juan Rojas, Phillip R. Musich, Zhiqiang Yao, Yong Jiang

**Affiliations:** ^1^ Department of Biomedical Sciences, J. H. Quillen College of Medicine, East Tennessee State University, Johnson City, TN, United States; ^2^ Center of Excellence in Inflammation, Infectious Disease and Immunity, James H. Quillen College of Medicine, East Tennessee State University, Johnson City, TN, United States; ^3^ Department of Internal Medicine, Division of Infectious, Inflammatory and Immunologic Diseases, Quillen College of Medicine, ETSU, Johnson City, TN, United States

**Keywords:** breast cancer, exosome, biomarker, TNBC, metastasis

## Abstract

Triple-negative breast cancer (TNBC) is a heterogeneous and invasive breast cancer (BC) subtype that is estrogen receptor-negative, progesterone receptor-negative, and human epidermal growth factor receptor 2 (Her2)-negative. So far, the treatment of TNBC is still ineffective due to the lack of well-defined molecular targets. Exosomes are nanosized extracellular vesicles composed of lipid bilayers. They originate from various types of donor cells and release a complex mixture of contents including diverse nucleic acid types (miRNA, LnRNA, siRNA, and DNA) and proteins; after binding to recipient cells the exosomes release their contents that execute their biological functions. Exosomes have been reported to play an important role in the tumorigenesis of TNBC, including tumor initiation, metastasis, angiogenesis, cell proliferation, immune escape, and drug resistance. On the other hand, exosomes can be valuable biomarkers for diagnosis, monitoring, and treatment of TNBC. More interestingly, exosomes can be harnessed as a nanosized drug-delivery system specifically targeting TNBC. In this review, we present the most recent mechanistic findings and clinical applications of exosomes in TNBC therapy, focusing on their use as diagnostic and prognostic biomarkers, nanoscale drug delivery platforms, and immunotherapeutic agents. In addition, the associated challenges and future directions of using exosomes for TNBC treatment will be discussed.

## Introduction

Breast cancer is one of the most common types of cancer among women in the world. According to published reports, breast cancer alone accounts for over 29% of all new cancer cases for women in 2022 (Siegel et al., 2016). However, triple-negative breast cancer (TNBC) accounts for approximately 15–20% of all breast cancer (Font-Clos et al., 2022). TNBC is characterized as estrogen receptor (ER) negative, progesterone receptor (PR) negative, and human epidermal growth factor receptor 2 (HER2) negative, while these receptors are commonly expressed in other subtypes of breast cancer. The typical properties of TNBC include its highly metastatic nature and heterogeneity, with limited treatment options leading to this being the deadliest subtype of breast cancer that often happens to young women (Perou et al., 2000; Sorlie et al., 2001; Haffty et al., 2006; Rakha et al., 2007). Currently, the major available treatment for TNBC is limited to chemotherapy; however, drug resistance and lack of well-defined and universal molecular targets seriously lower the overall survival probability of TNBC patients. Thus, the progress against TNBC requires further discovery of potential molecular targets and the development of an effective delivery system focusing on these targets. By now, the major therapy available for TNBC is chemotherapy with platinum (Pandy et al., 2019; Zhu et al., 2022) or untargeted chemotherapy (alone or in combination), providing limited choices with evident side effects (Medina et al., 2020; Mollah and Varamini, 2021). Hence, a safe and efficient targeted delivery system is in urgent need for TNBC therapy; in particular, development of more effective chemotherapic strategies will enable successful application of the antineoplastic drugs for TNBC treatment.

**FIGURE 1:**
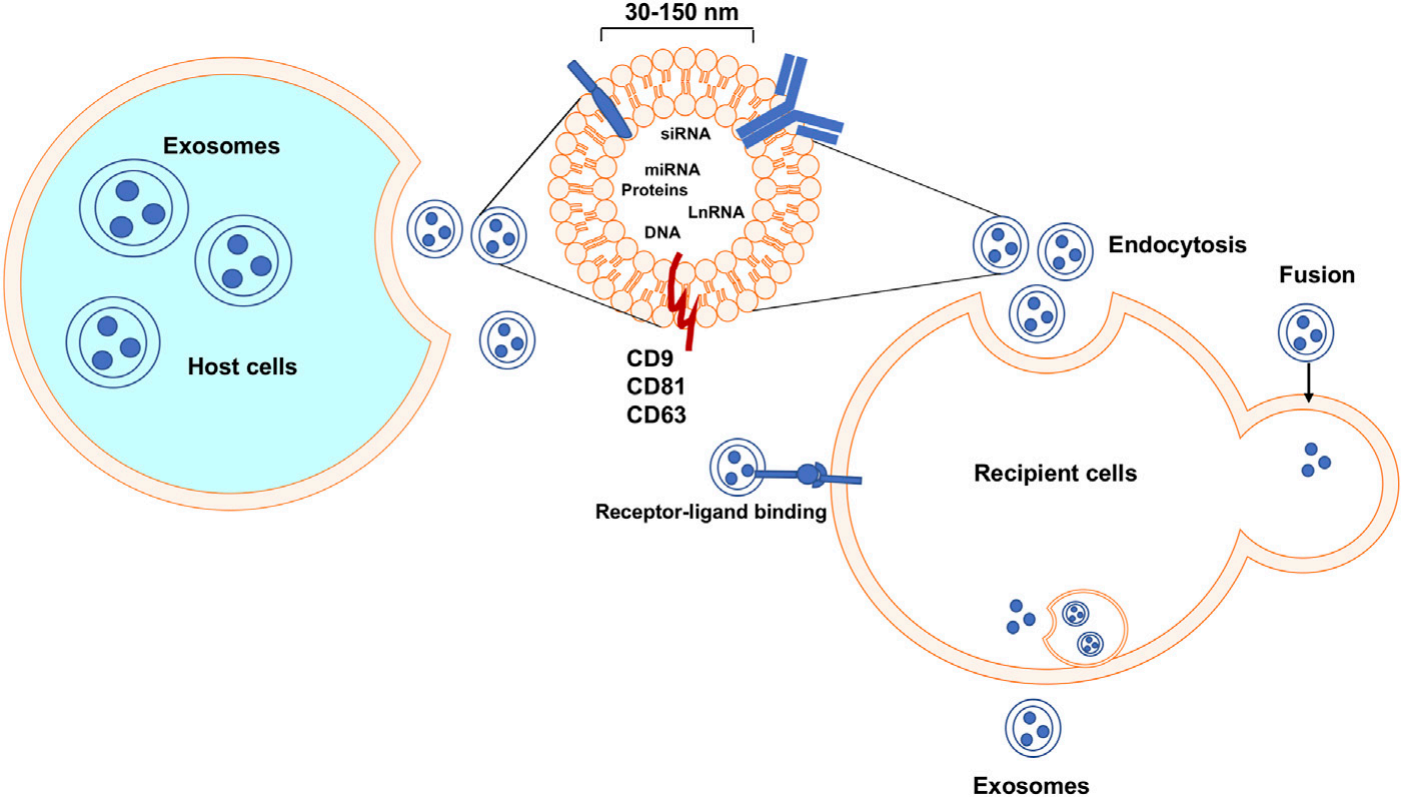
Exosomes biogenesis, secretion, uptake, and release of their contents. Exosomes are released after the fusion of a multivesicular body and cell membrane. Exosomes can enter recipient cells *via* multiple mechanisms and then may play essential roles in many molecular processes.

**FIGURE 2:**
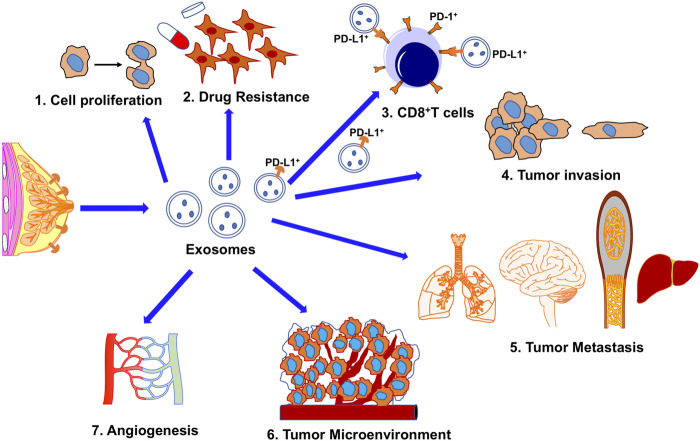
The potential roles of exosomes in TNBC biology and treatment stratagies. 1. Enhance tumor cell proliferation; 2. Increase drug resistance; 3. Immune-escape; PD-L1+ exosomes secreted from cancer cells confer acquired resistance to immunotherapy mediated by the direct binding between exosomal PD-L1 and T cells or between exosomes and α-PD-L1 monoclonal antibodies. 4. Stimulated tumor progression; 5. Metastasis; 6. Exosomes derived from cancer cells transfer contents to the tumor microenvironment; 7. Enhanced angiogenesis.

Exosomes are natural, nanoscale membrane vesicles secreted by almost all types of eukaryotic cells. Eberhard Trams and Rose Johnstone used the term “exosome” to define this group of extracellular vehicles (EVs) and in the 1980s determined their size to characterize the exosomes in the 1980s (Johnstone, 2005). Exosomes are lipid bilayer membrane vesicles with embedded transmembrane tetraspanin proteins including the common markers Cluster of Differentiation 9 (CD9), Cluster of Differentiation 63 (CD63), and Cluster of Differentiation 81 (CD81) (Shifrin et al., 2013; Colombo et al., 2014; Patton et al., 2015) (see Figure 1). These markers can be used for identification and classification of exosomes. Exosomes are reported to facilitate cell-cell communication through their engulfed functional biomolecules, such as proteins, RNA, DNA, lipids, *etc.* (Mathivanan et al., 2010; Higginbotham et al., 2016). As a kind of messenger carrier, a particular task of exosomes is to transmit various active biomolecules from host cells to their recipient cells; because they are cell-derived membranous structures exosomes can easily bind to and release their contents into another cell *via* membrane fusion (Figure 1). Exosomes are associated with the treatment of cancer due to their specific characteristics, especially when considering the ineffectiveness, severe immuno-repellence, and cytotoxicity of therapies employing some available synthetic therapeutic nanocarriers as the delivery agents (citations). Compared to other delivery systems, exosomes show very low immunogenicity, high biocompatibility, considerable efficacy, and very little, if any, accumulative toxicity in normal tissues. Thus, exosomes emerge as biologically natural nanocarriers for the targeted delivery of therapeutic agents to specific cells or tissues (Wang et al., 2020; Kucuk et al., 2021). One advantage of exosomes is their capability to be genetically engineered to carry therapeutic nucleic acids (miRNA, siRNA, and ncRNA) and proteins (Li et al., 2018). plus cell-surface. In addition, targeting molecules can be incorporated for specific cell surface proteins, as we propose in Figure 2, which is a project under development in our lab. Thus, these engineered exosomes derived from donor cells can deliver their contents to the recipient cells *via* specific fusion to their membranes or endocytosis. Biologically, exosomes and their released contents can modify physiological and pathological mechanisms in recipient cells. Furthermore, the major benefits of an exosome-based delivery system include specificity, safety, and biostability. Selective delivery of chemotherapeutic drugs *via* engineered exosomes into TNBC cells *via* ligand-receptor interactions on the cell surface and/or endocytosis to abolish drug resistance has a high potential to revolutionize treatment regimens and overcome current therapeutic dilemmas for TNBC (Kim et al., 2018).

## Exosome production and biological functions in TNBC

**FIGURE 3:**
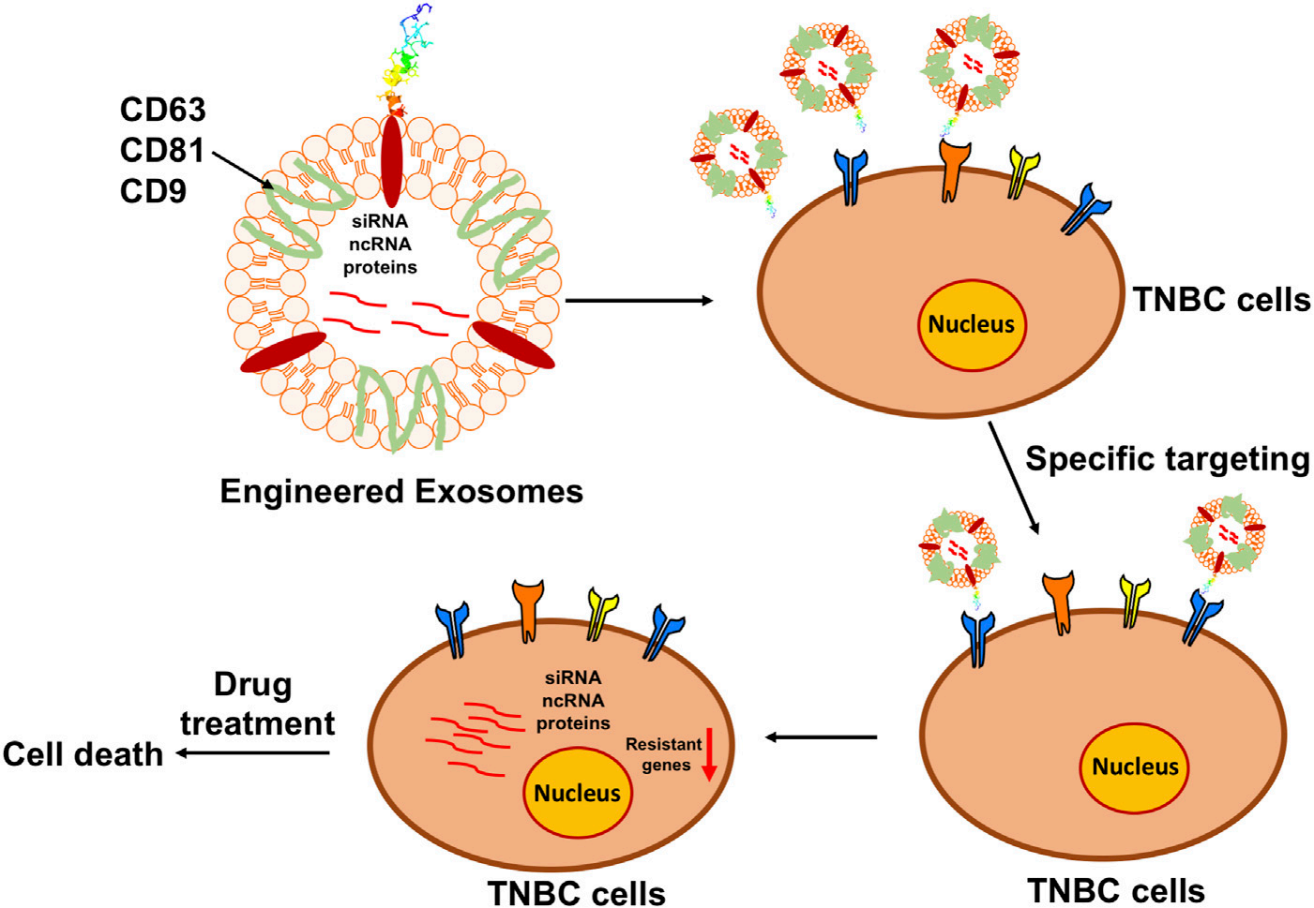
Diagram of the principle of engineering exosomes to facilitate chemotherapy. Based on the bioengineering techniques, the surface of exosomes can be modified with the designed ligands to precisely target the specific markers on the surface of TNBC cells, while the interior can be loaded with the functional anti-tumorigenesis cargoes to prevent the progression of TNBC.

Exosomes secreted from tumor cells, stromal cells, or immune cells can eventually change the tumor microenvironment to promote tumor progressions such as metastasis and drug resistance (Yousafzai et al., 2018). Exosomes have been considered the biological messengers for intercellular signaling that can transmit various biochemical and genetic information among cells. (Yousafzai et al., 2018). For example, it has been shown that after mRNAs carried by exosomes from the donor cells are taken up by the recipient cells, these mRNAs can be translated into proteins and secreted into the tumor microenvironment (Zhang et al., 2015). Numerous studies concluded that breast tumor cell-derived exosomes are involved in breast cancer initiation and progression (Yanez-Mo et al., 2015; Dioufa et al., 2017; Piombino et al., 2021), such as promoting cancer invasion and metastasis, accelerating angiogenesis, contributing to epithelial-mesenchymal transition (EMT), and enhancing treatment resistance in tumors (<*b*>Figure 3)</b>. Exosomal signaling can also facilitate the transfer of phenotypic characterization between TNBC cells (O'Brien et al., 2013). These suggest that exosomes isolated from TNBC cells can increase tumor invasiveness and further designate exosomes’ critical role in TNBC progression. Additionally, an exosomal protein, Rab27a, was found to play an essential role in promoting tumor progression and metastasis by facilitating the secretion of exosomes from TNBC cells (Wang et al., 2008). More importantly, some studies demonstrated that exosomal integrin expression could be used to predict target sites of tumor metastases (Hoshino et al., 2015). Extra studies further indicated that one part of the human epidermal growth factor receptor (HER) family was exosome-associated in breast cancer (Lowry et al., 2015). Recent studies also demonstrated that female breast cancer patients have a higher expression level of serum exosomal-annexin A2 (Exo-AnxA2) than healthy subjects, especially for TNBC, rather than luminal and HER2-positive breast cancer (Chaudhary et al., 2020). Conclusively, the dysregulation of exosomes has been closely correlated with the clinical-pathological characteristics and prognosis of TNBC patients (Tang et al., 2022). Considering the poor prognosis and lack of adequate response to conventional therapy of TNBC, the discovery of functional exosomes as a new marker for diagnosis of and therapeutic tool against TNBC should be a promising choice that can provide new opportunities for early diagnosis and improved clinical treatment of TNBC.

## Engineered exosomes as delivery vehicles for the therapy of TNBC

Different strategies have been employed for exosome-based treatment in TNBC. Currently, engineered exosomes are used as a therapeutic tool with demonstrated advantages due to their prolonged half-life in the circulation system besides low immunogenicity and low toxicity in both pre-clinical animal models and early clinical trials (Cully, 2021). Because of the lack of appropriate therapeutic targets, TNBC has been reported to have a poor prognosis, and clinical chemotherapeutics are limited. Therefore, the engineered exosomes become a candidate with great potential to improve the efficiency of drug delivery by enhancing their anti-tumor ability. As shown in <*b*>Table 1</b>, exosomes purified with S100 calcium-binding protein A4 (S100A4) play an essential role in tumor metastasis in hepatocellular carcinoma, demonstrated by both *in vitro* cellular models and *in vivo* mouse models (Sun et al., 2021). Furthermore, engineered exosomes, as a drug delivery platform, have been created to serve as the natural nanoparticles to carry S100A4 siRNA (siS100A4) for specifically targeting the pre-metastatic niche (PMN) of lungs, which has shown a significant suppressive effect on post-surgery metastasis in TNBC (Zhao et al., 2020). Gong et al. generated nanoscale target-specific exosomes (rich in metalloproteinase 15) that can specifically deliver cholesterol-modified miR-159 and doxorubicin (DOX) to TNBC cells. Exosomes co-loaded with DOX and cholesterol-miR159 have been shown to knock down the expression of the TCF-7 and effectively attenuated the proliferation of MDA-MB-231 TNBC cells in both *in vitro* and *in vivo* models (Gong et al., 2019). Macrophage-derived exosomes coated with poly (lactic-co-glycolic acid) displayed a high efficacy for targeted chemotherapy of TNBC (Mekseriwattana, et al., 2021). The precise targeting ability of the exosome-coated nanoparticles significantly increased the cellular uptake efficiency and displayed remarkable TNBC-targeting efficacy, resulting in cancer cell apoptosis and subsequent inhibition of cancer progression (Kim et al., 2018). As we know, erastin is a newly-developed chemotherapy drug that can instigate ferroptosis in TNBC cells; however, low water solubility and side effects undermined its application (Wu et al., 2019). To address this issue, Yu et al. loaded erastin into exosomes labeled with folate (FA) to target the TNBC cells overexpressing folate receptors on the cell surface, thereby enhancing ferroptosis with the pre-depletion of intracellular glutathione and overgeneration of reactive oxygen species (ROS), resulting in the significant attenuation of proliferative and migratory capabilities of MDA-MB-231 cells (Yu et al., 2019). Exosomes purified from mesothelin (MSLN)-targeted CAR-T cells robustly attenuate the growth of MSLN-positive TNBC cells. As a kind of cell-free treatment, exosomes derived from CAR-T cells show much lower toxicity and a higher effective tumor-inhibiting capability than living CAR-T cells (Yu et al., 2019; Yang et al., 2021). It has been found that the neutrophil-derived exosomes (N-Ex) decorated with superparamagnetic iron oxide nanoparticles (SPIONs) initiated tumor cell death by delivering cytotoxic proteins and activating the caspase-dependent apoptosis pathway to achieve a high tumor-targeting therapeutic effect in gastric cancer cells, colorectal cancer cells, and hepatic cancer cells, which were confirmed by both *in vitro* and *in vivo* models (Zhang et al., 2022). These engineered neutrophil-derived exosomes have not yet been tested for the treatment of TNBC, but they will be definitely a potential therapeutic candidate for TNBC therapy. Although many studies have indicated the potential clinical application of exosomes and there are many ongoing clinical trials with exosomes involved, so far no clinically exosome-based therapies have been approved yet. Taken together, it is evident from all pioneer studies imply that exosome is emerging as a novel promising option for TNBC treatment, and there is a high possibility of using engineered exosomes as multifunctional therapeutic agents for TNBC therapy. Moreover, in the future, we anticipate that exosomes from the TNBC tumor microenvironment can become the target for vaccination to improve TNBC therapeutic efficacy. However, in-depth scientific investigations are required to completely determine the safety and effectiveness of these exosome-based therapies and open new avenues for the better management of TNBC patients. Thus, comprehensive studies are required to develop a novel engineered exosome-mediated delivery platform to specifically target TNBC cells for effective and precise TNBC treatment with minimized off-target effects.

**TABLE 1:** Engineered exosomes in the treatment of TNBC and other cancers.

Specific targeting	Host cells	Recipient cells	Functions	References
S100 calcium-binding protein A (or its siRNA)	Hepatocellular carcinoma cells	Lung metastases	Activating (or preventing) STAT3 phosphorylation and up-regulating OPN expression	Sun et al. (2021); Zhao et al. (2020)
Disintegrin and metalloproteinase 15	Human monocyte-derived macrophage cells	Triple-negative breast cancer cells	Effectively silenced the TCF-7 gene and exhibited improved anticancer effects, without adverse effects	Gong et al. (2019)
Loaded with anticancer agent paclitaxel (PTX)	Macrophage cells	Pulmonary and liver metastases	Pulmonary metastases therapy	Kim et al. (2018)
Folate (FA) plus erastin	Human fetal lung fibroblasts	Triple-negative breast cancer cells	Enhancing ferroptosis and inhibiting the proliferation and migration of TNBC cells	Yu et al. (2019)
Mesothelin (MSLN)	Engineered CAR-T cells	Triple-negative breast cancer cells	Inhibited the growth of MSLN-positive TNBC cells without obvious side effects *in vivo*	Yu et al. (2019); Yang et al. (2021)
Superparamagnetic iron oxide nanoparticles (SPIONs)	Neutrophils from the peripheral blood of healthy donors	Human gastric cancer cells, colon cancer cells and liver cancer cells	Antitumor efficacy in xenograft tumor models	Zhang et al. (2022)

## Exosomes for immune escape in TNBC

Besides being used as a drug carrier, engineered exosomes also can be used for immunotherapy. Presently, immunotherapy has made appreciable progress against cancer. However, more efforts is needed to find a novel low-toxicity inhibitor and a highly specific delivery system with reliable biosafety. Exosomes are the prime delivery candidates due to their unique advantages described above. In the tumor microenvironment, exosomes can transfer bioactive molecules among tumor cells, immune cells, and stromal cells, which play a critical role to help cancer cells escape immune surveillance and acquire immune tolerance (<*b*>Figure 1 and</b > 3). Exosomes derived from CD8^+^ T lymphocytes are frequently involved in the modulation of various immune responses (Deenick et al., 2010; Chen et al., 2019). One of the mechanisms by which tumor cells escape immune surveillance is to upregulate PD-L1 expression on the cell surface. More and more experimental evidence shows that the overexpression of this extratemporal receptor has been implicated as a mechanism of resistance to immunotherapy, as illustrated in Figure 2. Pre-clinical *in vivo* studies have shown that PD-L1-carrying exosomes released by tumor cells can function as a systemic immunosuppressor in mice (Peinado et al., 2012; Chen et al., 2018; Poggio et al., 2019). Specifically, tumor cells can evade immunosuppression when exosome surface PD-L1 binds to the anti-PD-L1 antibody, leaving the tumor PD-L1 exposed, or when exosome surface PD-L1 binds to PD-1 on effector T cells during monoclonal antibody treatment (Chen et al., 2021). Therefore, the immunosuppressive activity of exosomes depends on exogenous PD-L1; this immunosuppression can be avoided by a pre-clear treatment of exosomes with an anti-PD-L1 antibody. Furthermore, exosomes enriched with PD-L1 serve as a favorable and predictive biomarker for immune checkpoint blockade (ICB) therapies for metastatic TNBC (Shen et al., 2019). In gastric cancer patients, a high level of PD-L1 carried by exosomes was correlated with decreased CD4+and CD8^+^ T cell infiltration shown by a reduction of granzyme B (GzmB) secretion in these cells (Chen et al., 2021).

Moreover, exosomes for immunotherapy in TNBC can be derived not only from lymphocytes but also can be from mesenchymal stem cells (MSCs), i.e. MSC-derived exosomes. MSC-derived exosomes also critically participate in immunomodulation, mainly by regulating the functions of immune cells and altering the secretion of inflammatory factors, such as TNF-α and IL-1β in inflammatory bowel disease (Liu et al., 2019). MSC-derived exosomes can facilitate the differentiation of monocytic myeloid-derived suppressor cells (M-MDSCs) into highly immunosuppressive M2-polarized macrophages. In addition, MSC-derived exosomes can impair protective anti-tumor immunity through the upregulation of PD-L1 in myeloid cells and the downregulation of PD-1 in T cells in breast cancer *in vivo* (Biswas et al., 2019). In pancreatic ductal adenocarcinoma, Bone marrow mesenchymal stem cell (BMSC)-derived exosomes carrying galectin-9 siRNA and oxaliplatin (OXA) prodrug can induce immunogenic cell death (ICD), and reverse the suppressive tumor immune microenvironment by inhibiting M2 macrophage polarization and promoting the recruitment of cytotoxic T lymphocytes, thus enhancing immunotherapy effectiveness *in vitro* and *in vivo* (Zhou et al., 2021). Therefore, exosome-mediated immunotherapy is a novel approach with great potential to revolutionize cancer therapy. This stems from the ability of exosomes to serve as carriers to instigate anti-cancer immune reactions and/or as a platform to deliver anti-cancer drugs.

## Clinical application potential and limitations of exosomes in TNBC

Exosomes carry tumor-specific cargoes and are very stable in the fluids circulating within the human body; therefore, as shown in Table 2, the detection of tumor exosomes is of significance for early diagnosis, evaluation of therapeutic efficacy, and prediction of treatment outcomes for cancer (Zhao et al., 2021). The results of Todorova et al., support previous reports in which plasma serves as a source of Exo-miRNA biomarkers in TNBC (Todorova et al., 2022). Zhong et al. showed that exosomal lncH19 was associated with poor clinical results in the serum of breast cancer patients, including positive lymph node metastasis and positive distant metastasis (Zhong et al., 2020). The Exo-miRNA signature in the plasma of patients with TNBC or HER2-positive breast cancer was significantly different from healthy controls, suggesting that exosomes might be useful in the diagnosis of breast cancer (Stevic et al., 2018) in addition to serving as effective delivery vehicles for tumor therapy. Moreover, Khan et al. demonstrated that survivin levels in exosomes from the sera of patients can be a diagnostic biomarker for TNBC (Khan et al., 2014). All these findings offer an exciting prospect for significantly in the clinical treatment of TNBC patients. In the context of TNBC, which is a genetic and biologically heterogeneous disease, the effects of exosomes on multiple targets may provide a novel therapeutic regimen for a combination therapy targeting several tumorigenesis-promoting pathways at one time (Liu et al., 2022). Translational medicine research of exosomes aiming to transform laboratory discoveries into novel clinical strategies to reduce TNBC incidence, morbidity, and mortality has been making excellent progress toward TNBC treatment by providing new regimens to discover diagnostic biomarkers and therapeutic strategies in TNBC therapy (Wang et al., 2021). Gong et al. developed a strategy to isolate exosomes exhibiting an elevated binding ability to integrin αvβ3. Binding occurred through a modified version of disintegrin and metalloproteinase 15 (A15) expressed on exosomal membranes (A15-Exo); this facilitated co-delivery of therapeutic quantities of Dox and cholesterol-modified miRNA 159 (Cho-miR159) to TNBC cells, both *in vitro* and *in vivo*. Also, as summarized in Table 2, some recent promising applications of exosomes in clinical TNBC treatment are under development. However, some limitations in the specific targeting of exosomes still remain. These include how to select the specific TNBC cell surface marker or receptor for targeting to exclude potential side effects, and how to design the proper exosome surface modules to maximize binding efficiency and specificity of the TNBC cell surface biomarker without damaging the exosome structure and content. In these design changes the engineered exosomes must retain their natural beneficial characteristics, Especially low immunogenicity. Although exosomes as a delivery system in clinical TNBC treatment has not been reported yet, their inherent abilities to cross the blood-brain barrier (BBB) and migrate from the bloodstream into the brain have been investigated for treating brain inflammatory disease *in vivo* (Alvarez-Erviti et al., 2011; Zhuang et al., 2011). Therefore, there is a high probability that exosomes can be modified to become an ideal targeted delivery carrier for clinical medication administration. Despite such a prospect for their broad application, in-depth research of exosomes is still required, including the development of a systematic engineering system for producing clinically applicable exosomes that can overcome the limitations of the current strategy.

**TABLE 2:** Clinical application of exosomes in TNBC.

Targets	Application	Source	Objective	References
Circulating Exosomal miRNAs	TNBC	Sera of TNBC patients	As biomarkers to predict treatment efficacy for TNBC as diagnostic tools	Todorova et al. (2022)
Exosomal miRNAs may modulate chemoresistance	TNBC	Sera of TNBC patients	Biomarkers of response to pembrolizumab in patients with TNBC	Stevic et al. (2019)
A15- Exo	TNBC	Human macrophages	A15- Exo to co-deliver miRNA and chemotherapeutics *via* biomimicry	Gong et al. (2019)
Proteomic profiling of exosomes	TNBC	Sera of TNBC patients	Circulating exosomal microRNA profiling was established for potential biomarkers and therapeutically targets identification	Zhao et al. (2018)
Exosomal miRNA profiles	TNBC	Sera of TNBC patients undergoing neoadjuvant chemotherapy	Establishing biomarkers	Aiko et al. (2021)
Survivin	Breast cancer	Sera of patients with breast cancer	Having early diagnostic value in cancer	Khan et al. (2014)

## Conclusion

Exosomes, as ideal drug carriers, have become attractive candidates for delivering therapeutic biomolecules to improve the therapy of solid tumors, including TNBC. Tumor metastasis and ineffective conventional treatments attribute TNBC to the poorest prognosis. Although recently some improvement in TNBC therapy has been achieved, it is still a serious challenge to discover novel options for the early diagnosis and prompt treatment of this kind of cancer. The important role of exosomes in many kinds of diseases has been well documented; however, only limited studies have focused on their critical role in TNBC disease and treatment. Thus, more research is required to explore the unprecedented utility of exosomes in TNBC therapy. Meanwhile, TNBC-derived exosomes have a high possibility to become a novel TNBC biomarker to diagnose TNBC *via* the specific proteins and nucleotides engulfed in exosomes; these exosomal molecules also can be the therapeutic targets for TNBC treatment if they function in tumorigenesis. In summary, although there are still some challenges, exosomes as a diagnostic marker and as a delivery platform for TNBC therapy will continue to unfold as a meaningful and promising prospect.
